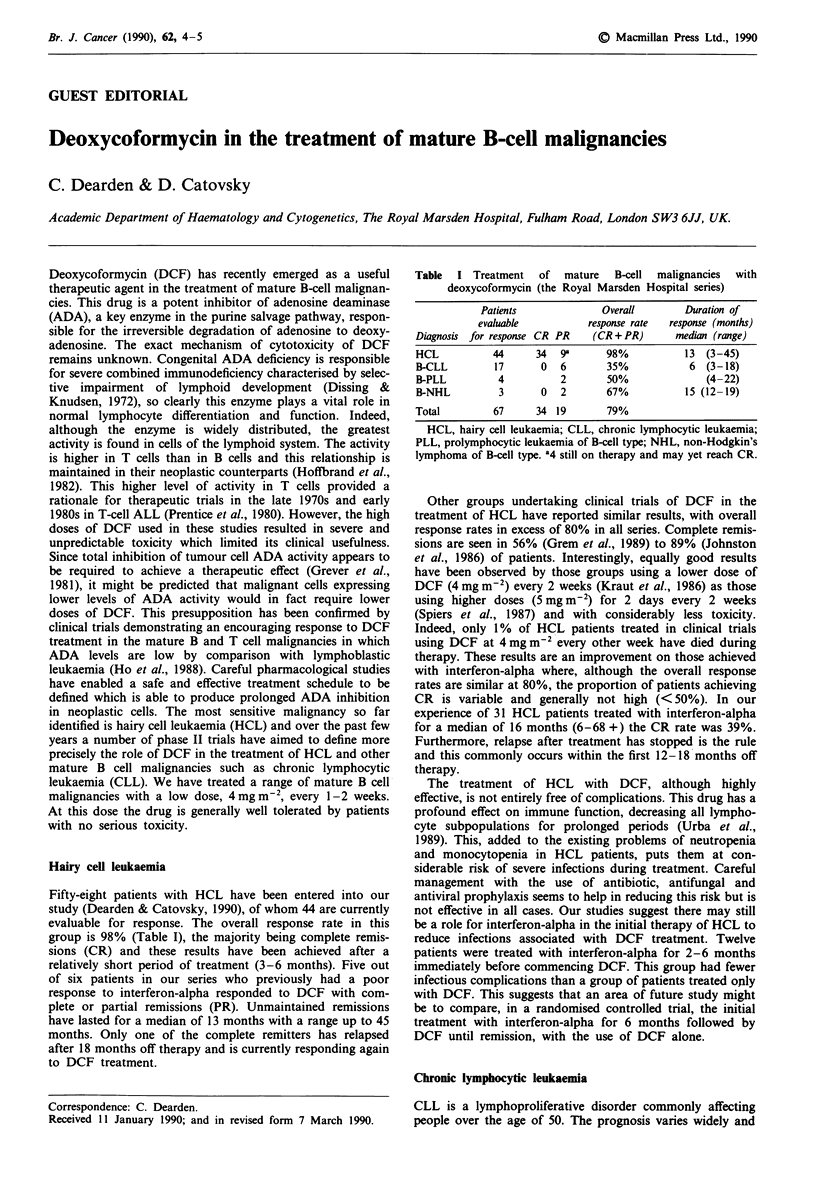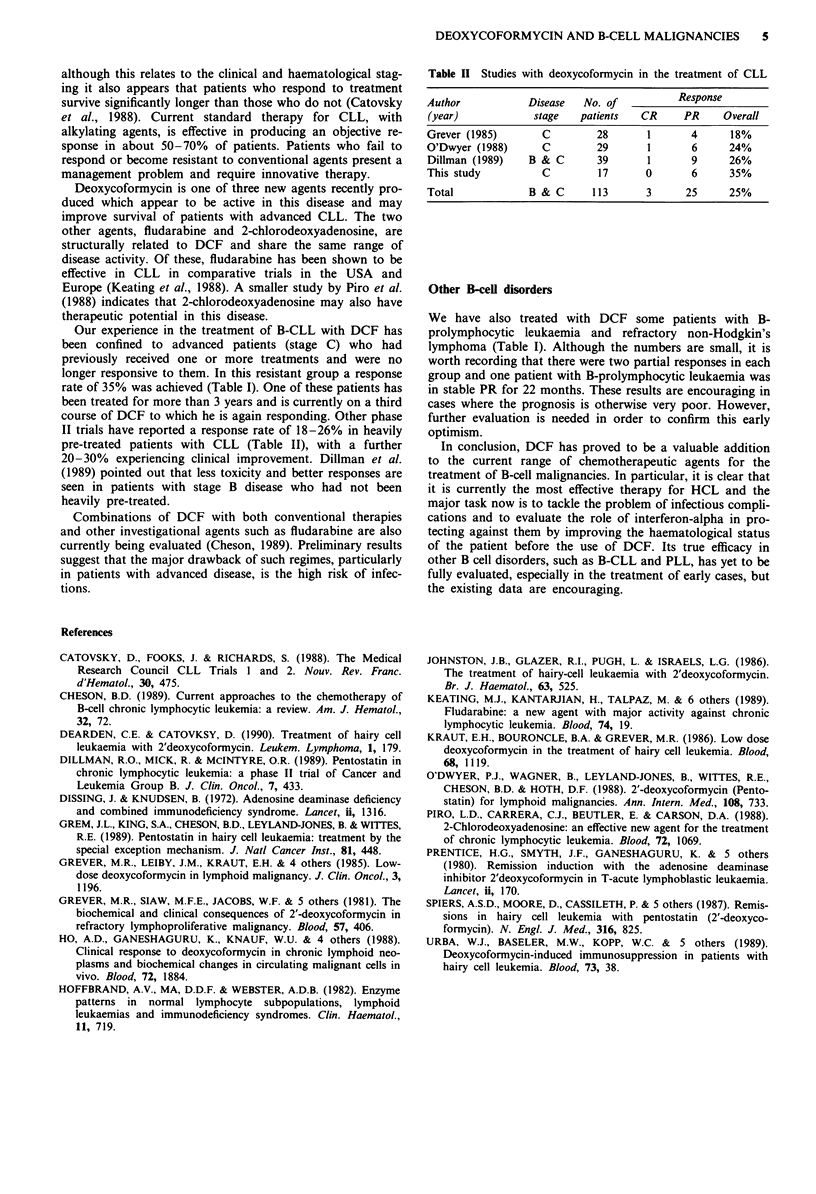# Deoxycoformycin in the treatment of mature B-cell malignancies.

**DOI:** 10.1038/bjc.1990.217

**Published:** 1990-07

**Authors:** C. Dearden, D. Catovsky

**Affiliations:** Academic Department of Haematology and Cytogenetics, Royal Marsden Hospital, London, UK.


Br. J. Cancer (1990), 62, 4-5 © Macmillan Press Ltd., 1990

GUEST EDITORIAL

### Deoxycoformycin in the treatment of mature B-cell malignancies

C. Dearden & D. Catovsky

*Academic Department of Haematology and Cytogenetics, The Royal Marsden Hospital, Fulham Road, London SW3 6JJ, UK.*

Deoxycoformycin (DCF) has recently emerged as a useful therapeutic agent in the treatment of mature B-cell malignancies. This drug is a potent inhibitor of adenosine deaminase (ADA), a key enzyme in the purine salvage pathway, responsible for the irreversible degradation of adenosine to deoxyadenosine. The exact mechanism of cytotoxicity of DCF remains unknown. Congenital ADA deficiency is responsible for severe combined immunodeficiency characterised by selective impairment of lymphoid development (Dissing & Knudsen, 1972), so clearly this enzyme plays a vital role in normal lymphocyte differentiation and function. Indeed, although the enzyme is widely distributed, the greatest activity is found in cells of the lymphoid system. The activity is higher in T cells than in B cells and this relationship is maintained in their neoplastic counterparts (Hoffbrand *et al.*, 1982). This higher level of activity in T cells provided a rationale for therapeutic trials in the late 1970s and early 1980s in T-cell ALL (Prentice *et al.*, 1980). However, the high doses of DCF used in these studies resulted in severe and unpredictable toxicity which limited its clinical usefulness. Since total inhibition of tumour cell ADA activity appears to be required to achieve a therapeutic effect (Grever *et al.*, 1981), it might be predicted that malignant cells expressing lower levels of ADA activity would in fact require lower doses of DCF. This presupposition has been confirmed by clinical trials demonstrating an encouraging response to DCF treatment in the mature B and T cell malignancies in which ADA levels are low by comparison with lymphoblastic leukaemia (Ho *et al.*, 1988). Careful pharmacological studies have enabled a safe and effective treatment schedule to be defined which is able to produce prolonged ADA inhibition in neoplastic cells. The most sensitive malignancy so far identified is hairy cell leukaemia (HCL) and over the past few years a number of phase II trials have aimed to define more precisely the role of DCF in the treatment of HCL and other mature B cell malignancies such as chronic lymphocytic leukaemia (CLL). We have treated a range of mature B cell malignancies with a low dose, 4 mg $m^{-2}$, every 1-2 weeks. At this dose the drug is generally well tolerated by patients with no serious toxicity.

### Hairy cell leukaemia

Fifty-eight patients with HCL have been entered into our study (Dearden & Catovsky, 1990), of whom 44 are currently evaluable for response. The overall response rate in this group is 98% (Table I), the majority being complete remissions (CR) and these results have been achieved after a relatively short period of treatment (3-6 months). Five out of six patients in our series who previously had a poor response to interferon-alpha responded to DCF with complete or partial remissions (PR). Unmaintained remissions have lasted for a median of 13 months with a range up to 45 months. Only one of the complete remitters has relapsed after 18 months off therapy and is currently responding again to DCF treatment.

Correspondence: C. Dearden.
Received 11 January 1990; and in revised form 7 March 1990.

**Table I** Treatment of mature B-cell malignancies with deoxycoformycin (the Royal Marsden Hospital series)

| *Diagnosis* | *Patients evaluable for response* | *CR* | *PR* | *Overall response rate (CR+PR)* | *Duration of response (months) median (range)* |
|---|---|---|---|---|---|
| HCL | 44 | 34 | 9[a] | 98% | 13 (3-45) |
| B-CLL | 17 | 0 | 6 | 35% | 6 (3-18) |
| B-PLL | 4 | | 2 | 50% | (4-22) |
| B-NHL | 3 | 0 | 2 | 67% | 15 (12-19) |
| Total | 67 | 34 | 19 | 79% | |

HCL, hairy cell leukaemia; CLL, chronic lymphocytic leukaemia; PLL, prolymphocytic leukaemia of B-cell type; NHL, non-Hodgkin's lymphoma of B-cell type. [a]4 still on therapy and may yet reach CR.

Other groups undertaking clinical trials of DCF in the treatment of HCL have reported similar results, with overall response rates in excess of 80% in all series. Complete remissions are seen in 56% (Grem *et al.*, 1989) to 89% (Johnston *et al.*, 1986) of patients. Interestingly, equally good results have been observed by those groups using a lower dose of DCF (4 mg $m^{-2}$) every 2 weeks (Kraut *et al.*, 1986) as those using higher doses (5 mg $m^{-2}$) for 2 days every 2 weeks (Spiers *et al.*, 1987) and with considerably less toxicity. Indeed, only 1% of HCL patients treated in clinical trials using DCF at 4 mg $m^{-2}$ every other week have died during therapy. These results are an improvement on those achieved with interferon-alpha where, although the overall response rates are similar at 80%, the proportion of patients achieving CR is variable and generally not high (<50%). In our experience of 31 HCL patients treated with interferon-alpha for a median of 16 months (6-68 +) the CR rate was 39%. Furthermore, relapse after treatment has stopped is the rule and this commonly occurs within the first 12-18 months off therapy.

The treatment of HCL with DCF, although highly effective, is not entirely free of complications. This drug has a profound effect on immune function, decreasing all lymphocyte subpopulations for prolonged periods (Urba *et al.*, 1989). This, added to the existing problems of neutropenia and monocytopenia in HCL patients, puts them at considerable risk of severe infections during treatment. Careful management with the use of antibiotic, antifungal and antiviral prophylaxis seems to help in reducing this risk but is not effective in all cases. Our studies suggest there may still be a role for interferon-alpha in the initial therapy of HCL to reduce infections associated with DCF treatment. Twelve patients were treated with interferon-alpha for 2-6 months immediately before commencing DCF. This group had fewer infectious complications than a group of patients treated only with DCF. This suggests that an area of future study might be to compare, in a randomised controlled trial, the initial treatment with interferon-alpha for 6 months followed by DCF until remission, with the use of DCF alone.

### Chronic lymphocytic leukaemia

CLL is a lymphoproliferative disorder commonly affecting people over the age of 50. The prognosis varies widely and although this relates to the clinical and haematological staging it also appears that patients who respond to treatment survive significantly longer than those who do not (Catovsky *et al.*, 1988). Current standard therapy for CLL, with alkylating agents, is effective in producing an objective response in about 50–70% of patients. Patients who fail to respond or become resistant to conventional agents present a management problem and require innovative therapy.

Deoxycoformycin is one of three new agents recently produced which appear to be active in this disease and may improve survival of patients with advanced CLL. The two other agents, fludarabine and 2-chlorodeoxyadenosine, are structurally related to DCF and share the same range of disease activity. Of these, fludarabine has been shown to be effective in CLL in comparative trials in the USA and Europe (Keating *et al.*, 1988). A smaller study by Piro *et al.* (1988) indicates that 2-chlorodeoxyadenosine may also have therapeutic potential in this disease.

Our experience in the treatment of B-CLL with DCF has been confined to advanced patients (stage C) who had previously received one or more treatments and were no longer responsive to them. In this resistant group a response rate of 35% was achieved (Table I). One of these patients has been treated for more than 3 years and is currently on a third course of DCF to which he is again responding. Other phase II trials have reported a response rate of 18–26% in heavily pre-treated patients with CLL (Table II), with a further 20–30% experiencing clinical improvement. Dillman *et al.* (1989) pointed out that less toxicity and better responses are seen in patients with stage B disease who had not been heavily pre-treated.

Combinations of DCF with both conventional therapies and other investigational agents such as fludarabine are also currently being evaluated (Cheson, 1989). Preliminary results suggest that the major drawback of such regimes, particularly in patients with advanced disease, is the high risk of infections.

**Table II** Studies with deoxycoformycin in the treatment of CLL

| *Author (year)* | *Disease stage* | *No. of patients* | *Response* CR | PR | Overall |
|---|---|---|---|---|---|
| Grever (1985) | C | 28 | 1 | 4 | 18% |
| O'Dwyer (1988) | C | 29 | 1 | 6 | 24% |
| Dillman (1989) | B & C | 39 | 1 | 9 | 26% |
| This study | C | 17 | 0 | 6 | 35% |
| Total | B & C | 113 | 3 | 25 | 25% |

### Other B-cell disorders

We have also treated with DCF some patients with B-prolymphocytic leukaemia and refractory non-Hodgkin's lymphoma (Table I). Although the numbers are small, it is worth recording that there were two partial responses in each group and one patient with B-prolymphocytic leukaemia was in stable PR for 22 months. These results are encouraging in cases where the prognosis is otherwise very poor. However, further evaluation is needed in order to confirm this early optimism.

In conclusion, DCF has proved to be a valuable addition to the current range of chemotherapeutic agents for the treatment of B-cell malignancies. In particular, it is clear that it is currently the most effective therapy for HCL and the major task now is to tackle the problem of infectious complications and to evaluate the role of interferon-alpha in protecting against them by improving the haematological status of the patient before the use of DCF. Its true efficacy in other B cell disorders, such as B-CLL and PLL, has yet to be fully evaluated, especially in the treatment of early cases, but the existing data are encouraging.

### References

CATOVSKY, D., FOOKS, J. & RICHARDS, S. (1988). The Medical Research Council CLL Trials 1 and 2. *Nouv. Rev. Franc. d'Hematol.*, **30**, 475.

CHESON, B.D. (1989). Current approaches to the chemotherapy of B-cell chronic lymphocytic leukemia: a review. *Am. J. Hematol.*, **32**, 72.

DEARDEN, C.E. & CATOVKSY, D. (1990). Treatment of hairy cell leukaemia with 2'deoxycoformycin. *Leukem. Lymphoma*, **1**, 179.

DILLMAN, R.O., MICK, R. & MCINTYRE, O.R. (1989). Pentostatin in chronic lymphocytic leukemia: a phase II trial of Cancer and Leukemia Group B. *J. Clin. Oncol.*, **7**, 433.

DISSING, J. & KNUDSEN, B. (1972). Adenosine deaminase deficiency and combined immunodeficiency syndrome. *Lancet*, **ii**, 1316.

GREM, J.L., KING, S.A., CHESON, B.D., LEYLAND-JONES, B. & WITTES, R.E. (1989). Pentostatin in hairy cell leukaemia: treatment by the special exception mechanism. *J. Natl Cancer Inst.*, **81**, 448.

GREVER, M.R., LEIBY, J.M., KRAUT, E.H. & 4 others (1985). Low-dose deoxycoformycin in lymphoid malignancy. *J. Clin. Oncol.*, **3**, 1196.

GREVER, M.R., SIAW, M.F.E., JACOBS, W.F. & 5 others (1981). The biochemical and clinical consequences of 2'-deoxycoformycin in refractory lymphoproliferative malignancy. *Blood*, **57**, 406.

HO, A.D., GANESHAGURU, K., KNAUF, W.U. & 4 others (1988). Clinical response to deoxycoformycin in chronic lymphoid neoplasms and biochemical changes in circulating malignant cells in vivo. *Blood*, **72**, 1884.

HOFFBRAND, A.V., MA, D.D.F. & WEBSTER, A.D.B. (1982). Enzyme patterns in normal lymphocyte subpopulations, lymphoid leukaemias and immunodeficiency syndromes. *Clin. Haematol.*, **11**, 719.

JOHNSTON, J.B., GLAZER, R.I., PUGH, L. & ISRAELS, L.G. (1986). The treatment of hairy-cell leukaemia with 2'deoxycoformycin. *Br. J. Haematol.*, **63**, 525.

KEATING, M.J., KANTARJIAN, H., TALPAZ, M. & 6 others (1989). Fludarabine: a new agent with major activity against chronic lymphocytic leukemia. *Blood*, **74**, 19.

KRAUT, E.H., BOURONCLE, B.A. & GREVER, M.R. (1986). Low dose deoxycoformycin in the treatment of hairy cell leukemia. *Blood*, **68**, 1119.

O'DWYER, P.J., WAGNER, B., LEYLAND-JONES, B., WITTES, R.E., CHESON, B.D. & HOTH, D.F. (1988). 2'-deoxycoformycin (Pentostatin) for lymphoid malignancies. *Ann. Intern. Med.*, **108**, 733.

PIRO, L.D., CARRERA, C.J., BEUTLER, E. & CARSON, D.A. (1988). 2-Chlorodeoxyadenosine: an effective new agent for the treatment of chronic lymphocytic leukemia. *Blood*, **72**, 1069.

PRENTICE, H.G., SMYTH, J.F., GANESHAGURU, K. & 5 others (1980). Remission induction with the adenosine deaminase inhibitor 2'deoxycoformycin in T-acute lymphoblastic leukaemia. *Lancet*, **ii**, 170.

SPIERS, A.S.D., MOORE, D., CASSILETH, P. & 5 others (1987). Remissions in hairy cell leukemia with pentostatin (2'-deoxycoformycin). *N. Engl. J. Med.*, **316**, 825.

URBA, W.J., BASELER, M.W., KOPP, W.C. & 5 others (1989). Deoxycoformycin-induced immunosuppression in patients with hairy cell leukemia. *Blood*, **73**, 38.